# Venezuelan Equine Encephalitis and Upper Gastrointestinal Bleeding in Child

**DOI:** 10.3201/eid1502.081018

**Published:** 2009-02

**Authors:** Stalin Vilcarromero, Alberto Laguna-Torres, Connie Fernández, Eduardo Gotuzzo, Luis Suárez, Manuel Céspedes, Patricia V. Aguilar, Tadeusz J. Kochel

**Affiliations:** Naval Medical Research Center Detachment, Lima, Peru (S. Vilcarromero, V.A. Laguna-Torres, P.V. Aguilar, T.J. Kochel); Hospital of Yurimaguas, Loreto, Peru (C. Fernandez); Cayetano Heredia University, Lima (S. Vilcarromero, E. Gotuzzo); General Directorate of Epidemiology, Lima (L. Suarez); National Institute of Health–Ministry of Health, Lima (M. Céspedes)

**Keywords:** VEE, arthropod-borne virus, febrile hemorrhagic diseases, encephalitis, gastrointestinal bleeding, Peru, Amazon Basin, dispatch

## Abstract

Venezuelan equine encephalitis (VEE) is reemerging in Peru. VEE virus subtype ID in Peru has not been previously associated with severe disease manifestations. In 2006, VEE virus subtype ID was isolated from a boy with severe febrile disease and gastrointestinal bleeding; the strain contained 2 mutations within the PE2 region.

Venezuelan equine encephalitis (VEE) is a reemerging disease in the Amazon region of Peru; subtype ID is the subtype most commonly isolated from humans (*1*–*3*). Although the typical clinical presentations of VEE are neurologic and febrile syndromes, in the Amazon region, the common presentation is a febrile syndrome with mild or no neurologic involvement (*1*–*4*). Human infections with VEE virus subtype IAB and IC produce more neurologic involvement and result in mortality rates as high as 0.5% during epidemics; however, epidemics with these viruses have not been reported in South America for more than a decade (*5*–*7*). During past epidemics, most neurologic disease and fatal cases were in children and elderly persons (*8*,*9*).

In 2006, an outbreak of febrile disease occurred in Jeberos, a rural Amazonian community in Loreto Peru, located 14 hours, by river, from Yurimaguas, Peru (Figure 1). Although VEE cases had been reported previously in Yurimaguas (T. Kochel, P.V. Aguilar, unpub. data), they had not been reported in Jeberos. We describe here the clinical manifestation of VEE subtype ID in a boy from Jeberos. The boy had severe disease; upper gastrointestinal bleeding; and neurologic, renal, and liver complications. He responded to supportive therapy.

**Figure 1:**
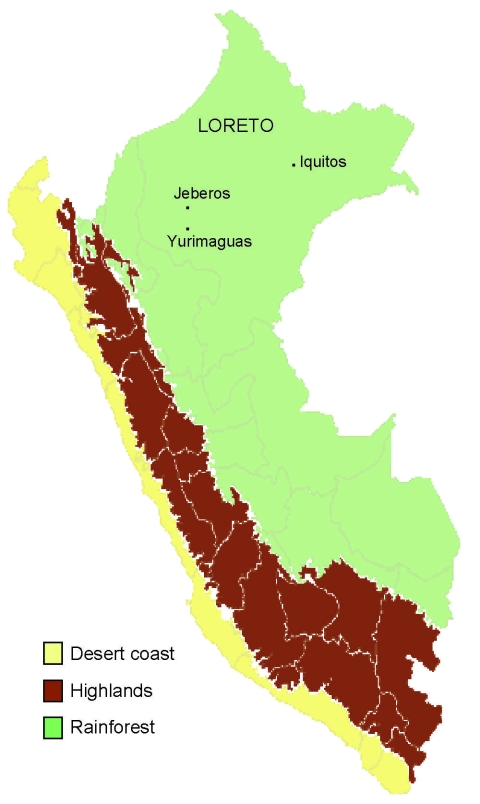
Map of Peru showing Jeberos community in Yurimaguas, Loreto.

## The Case

On January 9, 2006, a 3-year-old boy from Jeberos was admitted to the Hospital Santa Gema in Yurimaguas. Three days before hospitalization, he had fever, chills, and malaise. Two days before hospitalization, the symptoms persisted and the boy was brought by his parents to the Health Center of Jeberos, where he received antipyretic medication (acetaminophen and the nonsteroidal antiinflammatory drug metamizole) for fever (40°C). One day before hospitalization, the boy became sleepy and irritable and had 2 episodes of vomiting followed by melena. The boy was then transferred by airplane to the Hospital Santa Gema in Yurimaguas. On the day of hospitalization, the boy was in poor general condition and had coffee-ground emesis (with blood clots) and melena. His temperature was 37ºC, blood pressure was 90/60 mm Hg, respiratory rate was 45 breaths/min, and heart rate was 132 beats/min. He became somnolent, and marked paleness and scarce petechiae on both legs and severe dehydration were noted. No signs of jaundice, lymphadenopathy, or conjunctival bleeding were observed. No other signs were found in the thorax or cardiovascular system. Focal or meningeal signs and joint inflammation were absent.

Laboratory test results were as follows: leukocytes 12,200/mm^3^ (22% segmented cells, 0% bands, 75% lymphocytes, and 2% monocytes); platelets 122,000/mm^3^, hemoglobin 56 g/L, and hematocrit 17%. Liver function was altered: alanine aminotransferase (ALT) 132 U/L, aspartate aminotransferase (AST) 140 U/L, serum albumin 2.1 g/dL, and total bilirubin 2.4 mg/dL (direct bilirubin 1.4 mg/dL). Serum creatinine was 2.2 mg/dL and, because of the boy’s age, was considered acute renal failure. Preliminary diagnoses were febrile and hemorrhagic syndrome with severe dehydration and anemia. On the day of admission, January 9, a blood sample was sent to the Naval Medical Research Center Detachment in Lima, Peru, and the National Institute of Health–Ministry of Health of Peru, for advanced analysis.

The boy was given supportive therapy, antimicrobial drugs, and blood replacement (200 mL). On January 10, his leukocyte count had dropped to 4,200/mm^3^ (26% segmented, 14% bands, 56% lymphocytes, and 2% monocytes). After the transfusion, his hematocrit was 25%; his platelet count was 108,000/mm^3^ in the morning and decreased to 60,000/mm^3^ by night (Table). Test results for malaria (blood smear), agglutinins for typhoid fever, and surface antigen of hepatitis B virus were negative. The boy was hydrated and afebrile, but the melena and coffee-ground vomiting (with blood clots) persisted. On January 11, his hematocrit was 20% and platelet count was 91,000/mm^3^; he was still lethargic and the gastrointestinal bleeding persisted. On January 12, he received a transfusion of 150 mL whole blood; however, he still showed neurologic involvement (e.g., drowsiness, lethargy, confusion) and neck stiffness. On January 16, the boy’s condition improved and the gastrointestinal bleeding stopped. Liver and renal functions returned to within normal limits: serum creatinine 1.6 mg/dL, ALT 40 U/L, AST 18 U/L, urine volume 535 mL/24 h, and urine protein 36 mg/24 h. The boy’s mental status improved progressively, and he was discharged on January 20.

**Table:** Blood cell counts and hemoglobin values in a 3-year-old boy with Venezuelan equine encephalitis, Jeberos, Peru, January 2006*

Date/time of sample collection	Platelets/ mm^3^	Hematocrit, %	Leukocytes/ mm^3^	Leukocytes, %					Hemoglobin, g/L
				Neutrophils		Eosinophils	Monocytes	Lymphocytes	
				Bands	Segmented				
Jan 9	122,000	17	12,900	0	22	2	2	76	56
Jan 10									
4 h	108,000	25	4,200	14	26	2	2	56	
10 h	120,000	23	NM	NM	NM	NM	NM	NM	NM
16 h	87,000	22	NM	NM	NM	NM	NM	NM	NM
22 h	60,000	21	NM	NM	NM	NM	NM	NM	NM
Jan 11	91,000	20	NM	NM	NM	NM	NM	NM	NM
Jan 16	230,000	35	8,900	2	70	NM	NM	28	NM

*NM, not measured.

**Figure 2:**
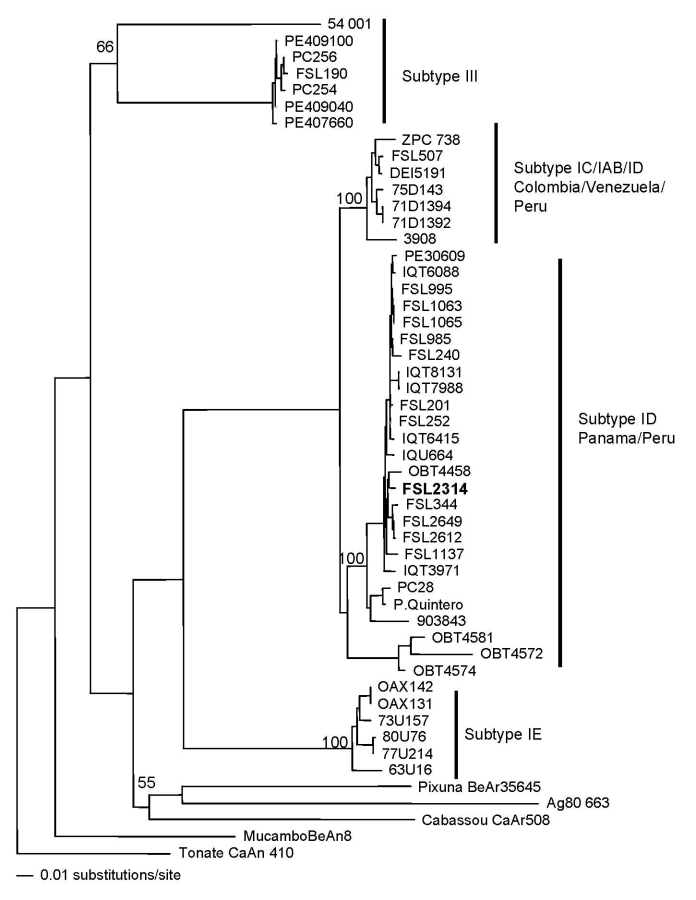
Neighbor-joining phylogenetic tree of Venezuelan equine encephalitis virus complex based on partial sequence of the envelope glycoprotein precursor segment. The strain isolated from the 3-year-old boy with upper gastrointestinal bleeding, Jeberos, Peru, January 2006, is shown in **boldface**. Numbers indicate bootstrap values.

VEE virus was isolated from serum culture in Vero cells. No other viruses were isolated in Vero or C6/36 cells. A convalescent-phase sample obtained ≈2 weeks after symptom onset had a >4-fold higher titer than the acute-phase sample, according to a previously described immunoglobulin (Ig) M ELISA specific for VEE (*2*). The sample was IgM negative for other local arthropod-borne viruses such as dengue, yellow fever, Mayaro, Oropouche, and members of the group C complex. Genetic analyses using previously described methods further identified the VEE strain as subtype ID, genotype Panama-Peru (*1*,*4*) (Figure 2). The strain was genetically similar to other strains isolated previously in Peru; however, 2 amino acid changes were observed within the envelope glycoprotein precursor (PE2) region (H→Q, V→I). The National Institute of Health–Ministry of Health reported negative results for leptospirosis and rickettsial diseases, according to IgM ELISA and indirect immunofluorescent assay, respectively.

In February 2006, another case of VEE in a child was reported. A 10-year-old boy with similar clinical signs, including headache, vomiting, melena, and altered mental status (somnolence and confusion) was admitted to the hospital in Yurimaguas. The boy, a resident of Esperanza village, Lagunas district, had visited the Aypena River near Jeberos. The acute-phase serum sample was positive for VEE virus by PCR; however, no virus was isolated in cell culture. A convalescent-phase sample, taken a week after onset of signs, was negative by VEE IgM ELISA; no additional convalescent-phase sample was obtained from the patient. No epizootics were reported.

## Conclusions

VEE virus subtype ID was isolated from a child with neurologic and severe hemorrhagic manifestations in the northern Peruvian jungle. During this episode, local health workers in Jeberos reported that they had evaluated at least 5 cases with some hemorrhagic manifestations; however, no samples were obtained from those cases, and thus there is no definitive proof that the patients were infected with VEE virus.

The VEE strain isolated from the boy with confirmed VEE contained 2 mutations within the PE2 region, which differed from other subtype ID strains from Peru. Nevertheless, the contribution of these or any other possible mutations in the viral genome to the hemorrhagic manifestation observed in the patient remains unknown. Many questions remain about the effect of VEE virus in the Jeberos community.
